# Evaluation of the serum levels of Mannose binding lectin‐2, tenascin‐C, and total antioxidant capacity in patients with coronary artery disease

**DOI:** 10.1002/jcla.23967

**Published:** 2021-09-07

**Authors:** Hamed Mehri, Naser Aslanabadi, Alireza Nourazarian, Behrouz Shademan, Fatemeh khaki‐khatibi

**Affiliations:** ^1^ Department of Biochemistry and Clinical Laboratories Faculty of Medicine Tabriz University of Medical Sciences Tabriz Iran; ^2^ Department of Heart and Artery Faculty of Medicine Tabriz University of Medical Sciences Tabriz Iran; ^3^ Department of Medical Biology Faculty of Medicine EGE University Izmir Turkey

**Keywords:** atherosclerosis, coronary artery disease (CAD), Mannose‐binding lectin (2), tenascin‐C, total antioxidant

## Abstract

**Background:**

Coronary artery disease (CAD) develops as a result of atherosclerosis. *Atherosclerosis* is a condition that leads to clogged arteries and can be caused by a variety of factors. Several studies have shown that various factors contribute to the development and progression of CAD. The aim of this study was to investigate the serum levels of MBL‐2, TNC and TAC in patients with CAD and the relationship between these biochemical parameters and the progression of CAD.

**Methods:**

In this study, 60 serum samples were obtained from CAD patients as the case group and 20 healthy serum samples as the control group. Serum levels of MBL‐2 and TNC were measured by the ELISA method. Serum TAC level was determined by calorimetry (spectrophotometry). In addition, MDA serum level was measured by reaction with thiobarbituric acid (TBA).

**Results:**

The mean age in the case and control groups was 58.4 ± 9.5 years and 85 ± 9.8 years, respectively. There was no significant difference in age, sex and family history in patients with CAD (*p* > 0.05), but there was a significant difference in blood pressure and smoking history (*p* > 0.05). Serum cholesterol, triglyceride, and LDL levels were significantly increased in the case group compared to the control group, while serum HDL‐C levels were significantly decreased in the case group. Serum levels of MBL‐2, TNC, and MDA were significantly increased in the case group compared to the control group. The serum level of TAC was significantly lower in the case group than in the control group.

**Conclusion:**

This study suggests that it is possible to diagnose patients with coronary artery disease (CAD) in the early stages of their disease and take preventive measures by measuring these parameters in serum. However, more research is needed before these serum parameters can be considered diagnostic biomarkers or therapeutic targets.

## Graphical abstract

**Background:** Coronary Artery Disease (CAD) develops as a result of atherosclerosis. Atherosclerosis is a condition that leads to clogged arteries and can be caused by a variety of factors. The aim of this study is to investigate the serum levels of MBL‐2, TNC and TAC in patients with CAD and the relationship between these biochemical parameters and the progression of CAD. **Methods:** In this study, 60 serum samples were obtained from CAD patients as the case group and 20 healthy serum samples as the control group. Serum levels of MBL‐2 and TNC were measured by the ELISA method. Serum TAC level was determined by calorimetry (spectrophotometry). In addition, MDA serum level was measured by reaction with thiobarbituric acid (TBA). **Results:** The mean age in the case and control groups was 58.4 ± 9.5 years and 85 ± 9.8 years, respectively. There was no significant difference in age, sex and family history in CAD patients (*p* > 0.05), but there was a significant difference in blood pressure and smoking history (*p* > 0.05). Serum levels of MBL‐2, TNC and MDA, were significantly increased in the case group compared to the control group. The serum level of TAC was significantly lower in the case group than in the control group. **Conclusion:** This study suggests that it is possible to diagnose patients with coronary artery disease (CAD) in the early stages of their disease and take preventive measures by measuring these parameters in serum. However, more research is needed before these serum parameters can be considered diagnostic biomarkers.

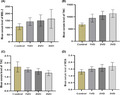

## 1 INTRODUCTION

Coronary artery disease (CAD) is caused by plaque buildup in the walls of the arteries that supply blood to the heart. Over time, the inside of the arteries narrows, a process called atherosclerosis.^1^ The prevention of coronary artery disease (CAD) and the associated reduction in mortality rates are significant concerns in developing countries.^2^, ^3^ In some individuals who are genetically prone to atherosclerosis or who consume excessive amounts of cholesterol and other lipids, significant amounts of cholesterol gradually accumulate in the arterial endothelium of the body. This condition is caused by the formation of atherosclerotic plaques that extend into the artery and obstruct all or part of the blood flow. Calcifications affect plaque homeostasis in several ways, including the properties of the calcifications themselves and their surrounding environment.^4^, ^5^ Angina pectoris and myocardial infarction are two diseases commonly associated with atherosclerosis of the coronary arteries.^6^

Lipids play an important role in atherosclerosis, and an increase in cholesterol levels is associated with accelerated disease progression. Elevated triglycerides may also be an independent risk factor for CAD, especially in women.^7^ On the other hand, high‐density lipoprotein (HDL‐C) seems to play a protective role and is inversely related to risk CAD.^8^

Many attempts have been made to treat or lower lipid levels, and research has shown that lowering cholesterol may help prevent primary and secondary coronary heart disease.^9^ Smoking has a significant impact on lipid profile and platelet activity and increases the risk of coronary artery disease (CAD) by 2 to 3 times.^10^, ^11^

There is some evidence for the role of Mannose binding lectin‐2 (MBL‐2) in the development of atherosclerosis. MBL‐2 is a member of the auxiliary cascade and plays an essential role in the first line of defense of the innate immune system against pathogenic microbes; since innate immunity is involved in atherogenicity, it has been postulated that MBL‐2 may be involved in the development of atherosclerotic plaques.^12^ A high prevalence of coronary artery disease (CAD) is associated with high MBL‐2 levels. Patients with and without coronary artery disease (CAD) who received an agent for atherosclerosis and periodontitis (PD) had lower serum MBL‐2 levels.^13^, ^14^

Elevated serum MBL‐2 levels are associated with the development of myocardial infarction and an unfavorable prognosis in coronary artery disease (CAD).^14^ Tenascin‐C (TNC) also plays an essential role in the development and progression of atherosclerotic plaques through cell migration and proliferation.^15^ In contrast, TNC promotes fibrosis and exerts repair effects on an experimental aneurysm through macrophage‐induced smooth muscle cell migration and proliferation.^16^ TNC enables adhesion to glycoprotein receptors on platelets to promote thrombosis in chronic atherosclerosis and is a highly complex molecule in the CAD process.^17^

Oxidative stress occurs when the amount of ROS produced is greater than the cell's ability to remove it.^18^ Malondialdehyde (MDA) is the end product of peroxidation of unsaturated fatty acids in cell membranes, which can be widely used in the diagnosis of oxidative stress.^19^ These substances may interact with lipoproteins, which are later absorbed by macrophages and contribute to the progression of atherosclerotic plaque and atherogenesis.^20^ In addition, plasma MDA levels were significantly elevated in individuals with hypertension, ischemic heart disease, cerebrovascular disease, or coronary artery disease.^21^ In addition, individuals who have had a heart attack have a decreased total antioxidant capacity (TAC), which predisposes them to coronary artery disease (CAD).^22^ The significance and originality of this study are that it investigates the diagnostic and predictive value of serum levels of Mannose binding lectin‐2, tenascin‐C and total antioxidant capacity in patients with coronary artery disease (CAD). This study mainly aims to investigate the serum levels of some biochemical markers such as MBL‐2, TNC, TAC, and MDA in patients with CAD to determine the relationship and dependence of these parameters on the severity of coronary artery disease. The aim is to investigate the possibility of using these parameters as biomarkers for early diagnosis before development CAD and prevention. Appropriate biomarkers are now considered an important tool for disease diagnosis. Therefore, serum levels of MBL‐2, TNC, TAC, and MDA can contribute to the diagnosis and evaluation of the treatment process of these patients. In addition, they can help in the development of treatment strategies to guide individualized treatment.

## 2 METHODS

### 2.1 Sample selection

This study is a cross‐sectional study approved by the Ethics Committee of the Faculty of Medicine, Tabriz University of Medical Sciences, with code 064/1398. For this study, 80 subjects were selected from Shahid Madani Hospital in Tabriz under the supervision of a cardiologist. A total of 60 patients were included in the case group (32 males and 18 females), and 20 patients (10 males and 10 females) were included as the control group. All patients in the case group had CAD symptoms, which were confirmed by angiography. Based on angiography results, patients were divided into three groups: 20 patients with one occluded vessel, 20 patients with two occluded vessels, and 20 patients with three occluded vessels. All patients gave informed consent. Information on blood pressure, smoking, family history, hyperlipidemia, age, and sex of the patients was collected by questionnaires (Table 1). The control group was selected from individuals who had no history of heart disease or other diseases and in whom angiographic findings were normal. Confounding factors were excluded as much as possible, such as individuals aged 40–70 years, individuals with diabetes (sugar above 120 mg/dl), and individuals taking hyperlipidemia medications and smoking.

**TABLE 1:** Clinical data of patient and control groups

Specifications	Control group	1VD	2VD	3VD
Gender (male/female) number	10/10	13/7	14/6	15/5
Age of the year	58.8 ± 9	59.5 ± 9	59.5 ± 9	5 ± 9
History of blood pressure number (%)	3 (15)	11 (55)	12 (60)	12 (60)
Family history number (%)	4 (20)	6 (30)	11 (55)	12 (60)
Cigarette smoking history number (%)	0(0)	8 (40)	11 (55)	10 (50)

Note

The case group with one clogged artery (1VD); the case group with one blocked artery (2VD); the case group with three clogged arteries (2VD).

### 2.2 Sampling

Ten ml of fasting blood was drawn from all subjects. All samples were collected between 8 and 11 am. Serum samples were separated from whole blood after clotting (60 min) by centrifugation (10 min at 1500 rpm). All experiments were performed in the biochemical laboratory of the Pharmaceutical Research Centre Tabriz University of Medical Sciences.

### 2.3 Biochemical experiments performed on serum samples

All tests for total cholesterol (Chol), triglycerides (TG), and HDL‐C were performed automatically using the Alcyon (the USA, model 300 Abbott) autoanalyzer. Serum total cholesterol concentration was measured by the enzymatic colorimetric (spectrophotometric) method and determined according to the instructions of the kit Pars Azmoun Company (CHOD‐PAP) at a wavelength of 492–550 nm.^23^ Serum concentration of TG was determined in mg/dl by Pars Azmoun kits, using the enzymatic GPO‐PAP method at a wavelength of 520–750 nm.^24^ Serum HDL‐C concentration and total cholesterol were measured by the enzymatic colorimetric method according to the instructions of the kit Pars Azmoun (CHOD‐PAP). However, according to the formula of William Friedwald, LDL‐C level can be determined by the concentration of cholesterol, triglycerides and HDL‐C: LDL‐C (mg/dl) = Chol ‐ (HDL‐C) _ TG/5.

### 2.4 Measurement of mannose binding lectin‐2 (MBL‐2) in serum

Mannose binding lectin‐2 measurement was based on biotin sandwich ELISA technology with a second antibody and according to the instructions of the kit Bioassay Technology (Cat. No.: E0211Hu). OD was measured at 450 nm for 10 min.

### 2.5 Measurement of tenascin‐C (TNC) in serum

Tenascin‐C was measured based on biotin secondary antibody sandwich ELISA technology and according to the instructions of the Bioassay Technology kit (Cat. No.: E1414Hu). OD was measured at 450 nm for 10 min.

### 2.6 Measurement of total antioxidant capacity (TAC) in serum

Total antioxidant capacity was measured colorimetrically (spectrophotometrically) according to the instructions of the Randox TAC kit. OD was measured at a wavelength of 600 nm. Serum samples without hemolysis were used to quantify laboratory conditions for total antioxidant status.

### 2.7 Measurement of Malondialdehyde (MDA) in serum

**FIGURE 1:**
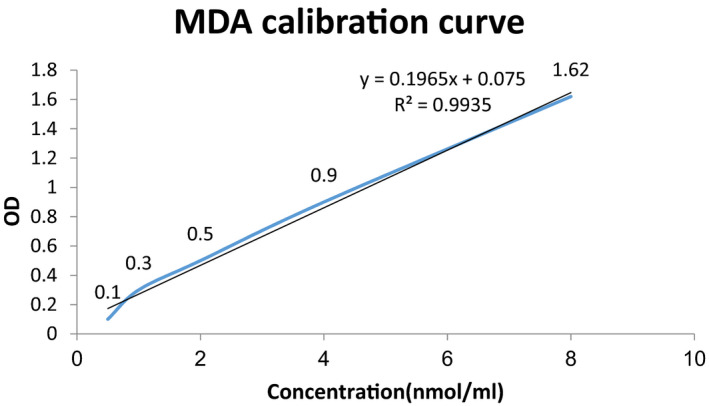
MDA calibration curve and its equation

The thiobarbituric acid (TBA) method was used to measure the MDA content in serum. Serum (500 L) was mixed with 1 ml of TBA (0.67%) and 3 ml of phosphoric acid (1%) and then kept in the bathroom for 45 min (Kumari and Menon, 1987). After cooling, the products were extracted in 3 ml of normal butanol and centrifuged at 3,000 rpm and 4°C for 10 min. The absorbance at 532 nm was determined using a spectrophotometer. The MDA calibration curve and the resulting equation are shown in Figure 1.

### 2.8 Statistical analysis

Statistical analysis was performed using SPSS software (version 16). The relationship between biochemical variables and indices was calculated based on Spearman's coefficient. An independent t‐test was performed for the comparison between the case group and the control group. One‐way analysis of variance (ANOVA) was used to compare the subgroups in the case group. Results were expressed as mean ± standard deviation. *p*‐value 0.05 was considered statistically significant.

## 3 RESULTS

### 3.1 Demographic characteristics of case and control groups

The present study was performed on 80 serum samples (60 patients and 20 controls). The mean age was 58.4 ± 9.5 years in the case group and 85 ± 9.8 years in the control group. There was no significant difference between the control and case groups in age, sex, and family history (*p* > 0.05). However, there was a significant difference in blood pressure (hypertension) and smoking history.

### 3.2 Serum lipid analysis in case and control groups

Serum cholesterol level was 184 ± 24 (mg/dl) in the studied patients. At the same time, this value was 160 ± 11.5 (mg/dl) in the control group. The different test results showed that the difference between the mean values in the two groups was statistically significant (*p* = 0.02). The mean TG value in the case group was 165 ± 34.5 (mg/dl), and the mean TG value in the control group was 136.5 ± 25 (mg/dl). Examination of the *t*‐test results showed that the difference between the mean TG values in the two groups was statistically significant (*p* = 0.04). The mean HDL‐C level in the case group was 33.5 ± 3 (mg/dl), and in the control group was 37 ± 3.4 (mg/dl). The results of the t‐test showed that the difference between the mean HDL‐C levels in the two groups was statistically significant (*p* = 0.02). The mean LDL‐C level was 118 ± 18 (mg/dl) in the case group and 101 ± 9.5 (mg/dl) in the control group. Examination of the *t*‐test results showed that the difference between the mean LDL‐C levels in the two groups was statistically significant (*p* = 0.02). Thus, serum cholesterol, LDL‐C, and TG levels were significantly elevated in the patient group compared with the control group. In contrast, serum HDL‐C levels were significantly decreased in the patient group (Table 2).

**TABLE 2:** Serum lipid profiles in patient and control groups

Variables	Control group (mean ± Sd)	1VD (mean ± Sd)	2VD (mean ± Sd)	3VD (mean ± Sd)	*p*‐Value
Chol (mg/dl)	160 ± 11.5	183 ± 23	181 ± 33	187 ± 26	0.02
TG (mg/dl)	136.5 ± 25	136.5 ± 25	166 ± 39.5	164.5 ± 36	0.04
HDL‐C (mg/dl)	37 ± 3.4	34 ± 3.3	34 ± 3.4	33 ± 2.3	0.02
LDL‐C (mg/dl)	101 ± 9.5	117 ± 15	115 ± 20	122 ± 18.5	0.02

Abbreviations: Chol, Cholesterol; HDL, High‐density lipoprotein; LDL, Low‐density lipoprotein; TG, Triglycerides.

### 3.3 Mannose Binding Lectin‐2 (MBL‐2) concentration levels in serum samples

**FIGURE 2:**
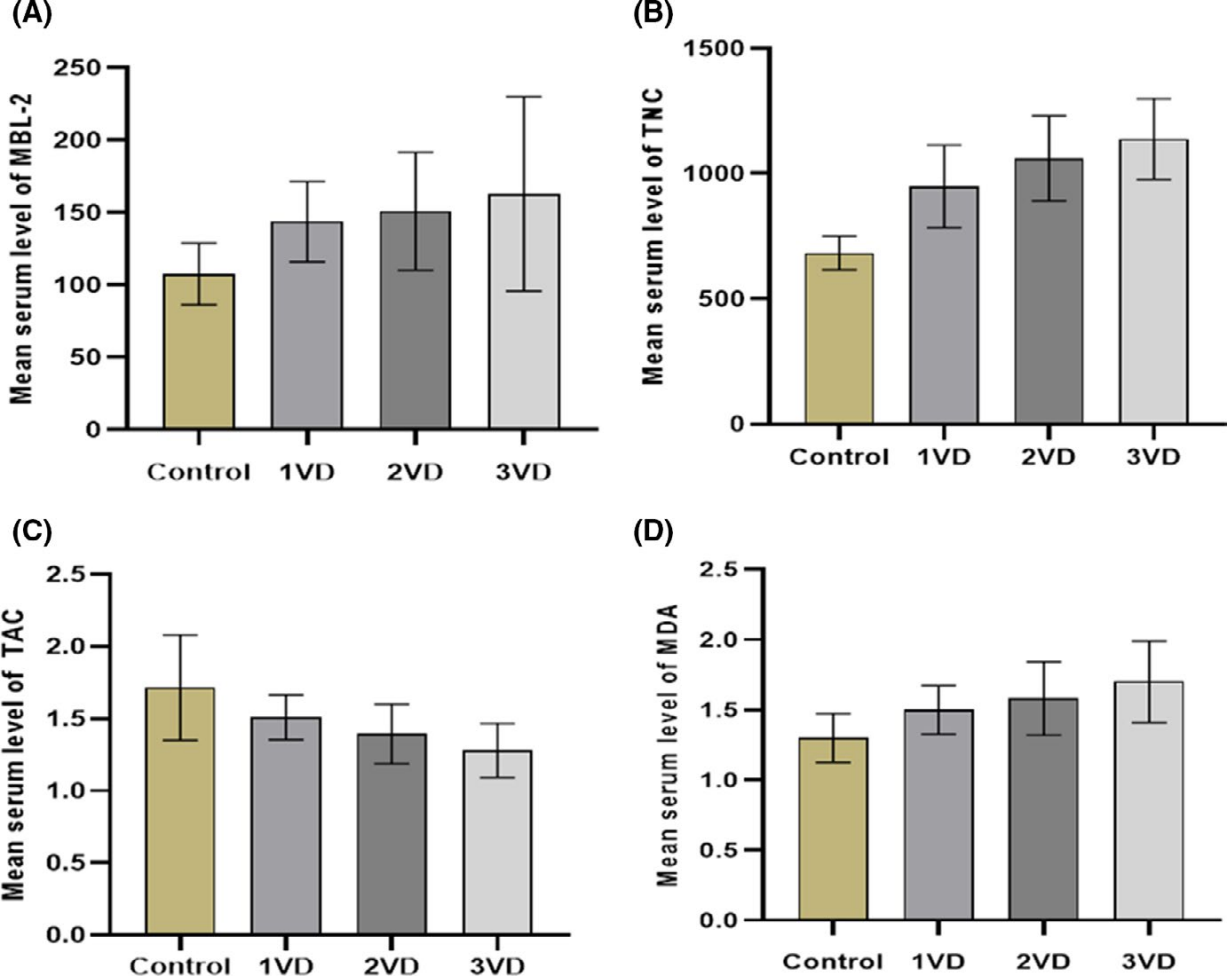
Mean serum level of MBL‐2 (A), TNC (B), TAC (C), and MDA (D) in the patient with coronary artery disease and control groups

The serum level of MBL‐2 was 152.5 ± 48 (ng/ml) in the case group and 108 ± 21 (ng/ml) in the control group. The results of the t‐test showed that the difference between the mean level of MBL‐2 in the two groups was statistically significant (*p* = 0.02). In other words, the serum level of MBL‐2 was significantly higher in the case group than in the control group (Table 3). Moreover, the results of the ANOVA test showed that the difference between the mean levels of MBL‐2 in the groups was statistically significant. The serum levels of MBL‐2 in the disease subgroups were also compared with the control group, and a significant difference was found between the two groups (*p* > 0.05) (Figure 2A).

**TABLE 3:** Mean serum level of MBL‐2, TNC, TAC, and MDA in the Patient and control groups

Variables	Control group (mean ± Sd)	Patient group (mean ± Sd)			*p*‐Value
		1VD	2VD	3VD	
MBL‐2 (ng/ml)	108 ± 21	152.5 ± 48			0.02
TNC (ng/ml)	683 ± 145	1049 ± 355			0.01
TAC (mmol/l)	1.7 ± 0.4	1.4 ± 0/2			0.01
MDA (nmol/ml)	1.3 ± 0.18	1.59 ± 0.25			0.01

Note

The case group with one clogged artery (1VD); the case group with one blocked artery (2VD); the case group with three clogged arteries (2VD).

### 3.4 Tenascin‐C (TNC) concentration levels in serum samples

The serum level of TNC was 1049 ± 355 (ng/ml) in the case group and 683 ± 145 (ng/ml) in the control group. As shown in Table 3, the difference between the mean value of TNC in the two groups was statistically significant (*p* = 0.01) (Table 3). Moreover, the ANOVA test results showed that the difference between the mean levels of TNC in the groups was statistically significant. The serum levels of TNC in the disease subgroups were also compared with the control group, and a significant difference was found between the two groups (*p* > 0.05) (Figure 2B).

### 3.5 Total Antioxidant Capacity (TAC) concentration levels in serum samples

In the case group, serum TAC level was 1.4 ± 0.2 (mmol/L), and in the control group, it was 1.7 ± 0.4 (mmol/L). The results of the *t*‐test showed that the difference between the mean value of TAC in the two groups was statistically significant (*p* = 0.01). In other words, serum TAC level was significantly increased in the case group compared with the control group (Table 3). The results of the ANOVA test showed that the difference between the mean values of TAC in the groups was statistically significant. The serum levels of TAC in the disease subgroups were also compared with the control group, and a significant difference was found between the two groups (*p* > 0.05) (Figure 2C).

### 3.6 Malondialdehyde (MDA) concentration levels in serum samples

As shown in Table 3, the serum MDA level was 1.59 ± 0.25 (nmol/ml) in the case group and 1.3 ± 0.18 (nmol/ml) in the control group. The results of the *t*‐test showed that the difference between the mean values of MDA in the two groups was statistically significant (*p* = 0.01) (Table 3). In addition, the results of the ANOVA test showed that the difference between the mean MDA values of the two groups was statistically significant. In addition, the serum MDA levels in the disease subgroups were compared with the control group, and a significant difference was found between the two groups (*p* > 0.05) (Figure 2D).

## 4 DISCUSSION

Coronary artery disease is a significant problem all over the world. Some studies have been done to diagnose this problem and prevent it from being solved with the least harm. Many studies suggest that there are proportional differences in serum concentrations of various lipids between the CAD and control groups, which may contribute to the development and progression of CAD.^25^, ^26^ A significant difference in chol, TG and LDL‐C concentrations was found in the control group and CAD patients in one study, and a significant decrease in HDL‐C levels was observed in CAD patients.^27^

In this study, there was a significant increase in chol, TG and LDL‐C levels in patients with CAD and a significant decrease in HDL‐C levels, which is consistent with previous research findings in this area.^28^ HDL particles play an important role in reverse cholesterol transport, the process by which atheroma can be reversed. HDL particles scavenge cholesterol from foam cells and reduce cholesterol oxidation and future foam cell development. In this way, the vicious cycle can be broken, and inflammation of the arterial wall can be prevented or reduced. HDL particles can also restore the normal function of endothelial cells and suppress inflammation, chemotactic and thrombosis.^29^ In this way, decreased HDL in patients with CAD may not prevent the likelihood of cholesterol oxidation and the development of more foam cells. Therefore, a decrease in HDL may be one of the factors contributing to the development of atherosclerosis in these individuals.

The relationship between MBL‐2 and atherosclerotic disease is controversial. It remains mainly unknown.^14^ However, in a large prospective study of apparently healthy individuals, high‐serum MBL‐2 levels in men were associated with a higher risk of developing coronary heart disease in future. This association was independent of cardiovascular risk factors. This suggests that MBL‐2 levels reflect or contribute to a pathophysiological process associated with the development of atherosclerosis. In addition, there is evidence for the role of MBL‐2 in CAD.^30^ The results of our study showed that the serum level of MBL‐2 was significantly increased in patients with coronary artery disease compared to the control group. It is also related to disease severity, with higher serum MBL‐2 levels observed in individuals whose arteries are more severely affected. These results suggest that MBL‐2 may play a role in the development of atherosclerotic plaques in arteries.^30^

TNC is involved in both physiological regeneration and the pathology of blood vessels.^31^ Under normal circumstances, small amounts of TNC are found in adult tissues. However, upregulation of TNC has been found in wound healing, cancer development, and cardiovascular disease, where expression levels of TNC appear to be a reliable indicator of disease progression and poor prognosis.^32^ Wallner et al. examined TNC expression in human coronary atherosclerotic plaques obtained from heart transplant patients and showed that TNC immunization was higher in the ruptured area and that the level of TNC expression was associated with inflammation.^33^

In 2014, Sakamoto et al.^34^ reported the expression and accumulation of TNC in arterial wall damage contributing to plaque inflammation and rupture. These data demonstrate that TNC is related to plaque instability and thrombogenicity after plaque rupture in acute coronary syndrome. TNC interaction with the Rho GTPase pathway may be a functional mechanism by which TNC can promote SMC migration and proliferation essential for the development of atherosclerotic plaques.^15^ In our study, serum levels TNC were elevated in CAD patients compared to the control group. The results suggest that elevated TNC levels lead to thrombogenic plaque formation. TNC causes thrombosis by increasing the adhesion of platelets to each other. Free radicals (anions superoxide and hydroxyl radicals) are formed by reducing oxygen in the body and can damage all biomolecules.^35^ Under normal conditions, these free radicals are neutralized by the immune system and antioxidants in the blood and tissues.^36^ An increase in oxygen free radicals has been reported in myocardial infarction. Increased oxidative stress and defects in the antioxidant defense system play an important role in endothelial dysfunction and are considered significant factors in the progression of atherosclerosis.^37^ MDA is the end product of lipid peroxidation and one of the most efficient and comprehensive indicators of oxidative stress.^38^ Therefore, MDA is considered a reliable indicator of oxidative damage. In one study, the relationship between oxidative stress and atherosclerosis was determined by measuring MDA levels.^39^ In addition, serum MDA concentrations were measured, and MDA levels were significantly higher in the CAD group than in the control group.^40^ In another study, high MDA levels were found in patients with atherosclerosis compared to the control group.^22^ The results showed that serum MDA level was significantly increased in the coronary artery disease group compared to the control group, confirmed by previous studies. Thus, an increase in free radicals may increase MDA, which may play a significant role in increased atherosclerosis. According to some findings, the number of narrowed arteries in patients with chronic coronary artery disease may be positively associated with an increased oxidative state. As the number of narrowed arteries increases, the amount of oxidative stress to which these individuals have been exposed increases proportionally.^40^

Research on the relationship between antioxidants and CAD is conflicting. In the study by Bagheri et al., patients with CAD had higher TAC levels than the control group.^41^ A research group measured higher TAC levels in people with hypertension than controls using the OXY adsorption test. Their HDL cholesterol was low, and TAC was significantly related to the prevalence and severity of CAD. In addition, patients with atherosclerosis were found to have higher TAC levels than healthy individuals.^42^ Some studies have shown that antioxidants and blood TAC in patients with CAD.^27^

Previous studies have provided confounding results regarding the association between cardiovascular disease and TAC. One reason for the lack of a significant association or increase in TAC in patients with CAD could be differences in TAC measurement methods. In a study of healthy smokers and nonsmokers, an increase in free radicals was found in smokers compared with nonsmokers.^37^ However, in our study, serum TAC levels decreased in coronary artery disease, indicating the role of TAC in this disease.^43^ The present study results indicate that serum MDA levels are significantly increased in smokers compared with nonsmokers with CAD, so smoking may also play a role in atherosclerosis. This investigation was subject to several limitations. First, it was a study at a single center with a limited number of inpatients. The patient population of a hospital is the only thing that can be considered in these statistics. The number of outpatients with relatively stable conditions is also essential, and their conditions may differ from those of inpatients. As a result, we are limited in assessing the correlation between biochemical biomarkers and coronary artery disease. Given the limited number of samples, it is recommended that results be interpreted with caution. Therefore, further results are needed to reach a reliable conclusion. Second, long‐term follow‐up data are lacking, so future research with a larger sample size and long‐term follow‐up data are needed. Third, obesity is an independent risk factor for coronary artery disease (CAD) related to recurrent fat abnormalities (recurrent fat deposits). It is recommended that information on obesity be included in future studies.

## 5 CONCLUSION

Studies have also shown that individuals with coronary artery disease had higher serum levels of MBL‐2, TNC, and MDA but lower serum levels of TAC. It is possible to measure these parameters in the serum of patients to detect the disease earlier and take preventive measures. Moreover, by analyzing the advanced stages of CAD, appropriate treatment strategies can be applied. However, further studies are needed to consider these cases as follow‐ups to treatment targets or procedures. These biomarkers may affect different groups of CAD.

## CONFLICTS OF INTEREST

The authors declare that there are no conflicts of interest.

## AUTHOR CONTRIBUTIONS

HM, NA, and FKH were involved in study design; BSH and HM contributed to analysis and interpretation of data; AN and FKH contributed to revising the manuscript content; and AN and FKH gave consent for the final version of the manuscript.

## Data Availability

All data obtained in the study can be accessed if desired.
